# Systemic Erysipelas Outbreak among Free-Ranging Bottlenose Dolphins, San Diego, California, USA, 2022

**DOI:** 10.3201/eid2912.230811

**Published:** 2023-12

**Authors:** Kerri Danil, Kathleen M. Colegrove, Martha A. Delaney, Alexandria Mena, Nancy Stedman, Elyse Wurster

**Affiliations:** Southwest Fisheries Science Center, La Jolla, California, USA (K. Danil);; University of Illinois, Brookfield, Illinois, USA (K.M. Colegrove, M.A. Delaney);; SeaWorld, San Diego, California, USA (A. Mena);; Busch Gardens, Tampa, Florida, USA (N. Stedman);; Ocean Associates Inc., under contract to Southwest Fisheries Science Center, La Jolla (E. Wurster)

**Keywords:** Erysipelas, Erysipelothrix rhusiopathiae, sepsis, mammals, dolphins, bottlenose dolphins, Tursiops truncatus, zoonoses, animal diseases, environment, bacteria, Pacific Ocean, California, United States

## Abstract

We diagnosed fatal *Erysipelothrix rhusiopathiae* sepsis in 3 stranded bottlenose dolphins (*Tursiops truncatus*) during summer 2022, in San Diego, California, USA. The previously undetected disease in this relatively small, regional population of dolphins most likely indicates an environmental or biological change in the coastal ocean or organisms.

Erysipelas is a disease of animals caused by the bacterium *Erysipelothrix rhusiopathiae*, which can be transmitted via exposure to feces, urine, saliva, and nasal secretions from infected animals and contaminated food, water, and soil (*1*). Human infection with this bacterium most often involves occupational exposure (*1*). In cetaceans, the disease is thought to be caused by ingesting infected fish, tooth raking from infected conspecifics, or infected wounds. Chronic cutaneous and acute fatal septicemic forms of the disease have been reported for captive and free-ranging cetaceans (*2*) but not for free-ranging cetaceans along the Pacific Coast of the United States.

Two stocks of bottlenose dolphins (*Tursiops truncatus*) inhabit the waters of California, USA: coastal and offshore. The coastal population comprises ≈500 dolphins that range from San Francisco, California, USA, to San Quintin, Mexico (latitudinal distance = 802 km), with little site fidelity (*3*). In southern California, the coastal bottlenose dolphins are typically found within 500 meters of the land.

During summer 2022 (June–September), 3 coastal bottlenose dolphins, of mixed sex and age class, were found stranded within 46 km of each other in San Diego, California, USA; we diagnosed sepsis caused by *E*. *rhusiopathiae*. The diagnoses coincided with increased strandings for this species in the region. In 2022, a total of 8 bottlenose dolphins were stranded, compared with a 20-year average of 4.35 per year (K. Danil, unpub. data; calculated by using Southwest Fisheries Science Center stranding records).

We determined cause of death for 6 of the 8 dolphins: 3 systemic erysipelas, 1 brucellosis, 1 trauma, and 1 malnutrition (Table). Gross necropsy findings for the 3 with erysipelas included open rake wounds (Appendix), mottled livers, distended urinary bladders, empty stomachs, and pulmonary edema; 2 dolphins also had ascites and icterus. Histopathologic examination for the 3 dolphins with erysipelas indicated vasculitis associated with multiorgan inflammation, necrotizing adrenalitis and nephritis for 1, and gastroenteritis for 1. Intracellular bacteria were identified (Figure), and *E. rhusiopathiae* were cultured from >2 organs from all 3 dolphins (Table). We confirmed the identity of all colonies of interest by using biochemical testing and matrix-assisted laser desorption/ionization time-of-flight mass spectrometry. Overall, the gross and histopathologic findings were consistent with other reports of *E. rhusiopathiae* infection in cetaceans (*2*).

**Table:** Characteristics of *Tursops truncatus* dolphins stranded in San Diego, California, USA, 2022

Specimen	Latitude and longitude	Decomposition status	Sex	Strand date	Age class	*Erysipelothrix* *rhusiopathiae* culture	Cause of death
KXD0391	32.8764, −117.2513	Fresh	F	May 16	Neonate	Spleen –, lung –, liver –	*Brucella* infection
**KXD0393**	**33.05023, −117.2995**	**Fresh**	**F**	**Jun 26**	**Juvenile**	**Brain +, spleen +**	***Erysipelothrix* sepsis**
KXD0394	32.6404, −117.146	Moderate	M	Jun 28	Neonate	NA	Trauma
**KXD0395**	**32.9055, −117.2555**	**Fresh**	**F**	**Jul 2**	**Adult**	**Kidney +, spleen +**	***Erysipelothrix* sepsis**
SWC-TT-2201B	32.5789, −117.1323	Fresh	F	Aug 29	Neonate	Spleen –	Malnutrition
KXD0399	32.7095, −117.2349	Advanced	F	Sep 11	Adult	NA	Unknown, no necropsy
**SWC-TT-2202B**	**32.6398, −117.1463**	**Fresh**	**M**	**Sep 12**	**Calf**	**Brain +, spleen +, lung +**	***Erysipelothrix* sepsis**
KXD0400	32.8606, −117.2559	Advanced	F	Sep 15	Juvenile	NA	Unknown, no necropsy

*Boldface indicates erysipelas cases. NA, not applicable; +, positive culture result; –, negative culture result.

**Figure:**
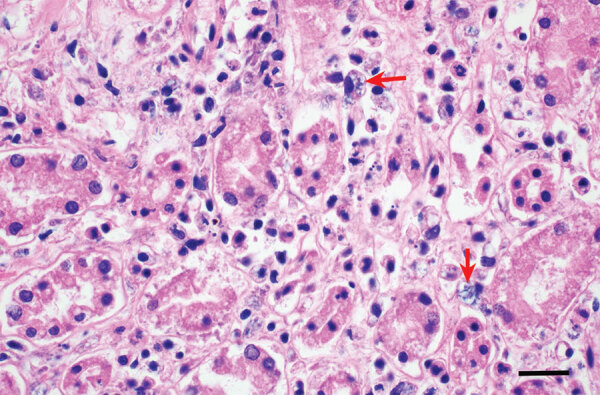
Section of kidney with neutrophilic nephritis associated with histiocytic bacterial rods (arrows) consistent with *Erysipelothrix* infection in specimen KXD0395 from study of systemic erysipelas outbreak among free-ranging bottlenose dolphins, San Diego, California, USA, 2022. Hematoxylin and eosin stain; scale bar indicates 20 microns.

For the past 20 years, histopathology and microbiology have been used to determine marine mammal cause of death in the San Diego region. Lack of erysipelas detection during that time suggests that the recent cluster indicates emerging erysipelas in cetaceans of this region. Similarly, a recent large mortality event of harbor porpoises (*Phocoena phocoena*) in the Netherlands was attributed to *E. rhusiopathiae*, which had not been previously detected in that area (*4*). The close temporal and geographic proximity of the affected dolphins in San Diego suggests that an erysipelas outbreak may have led to the increased coastal bottlenose dolphin deaths in this region. Although the causes of this outbreak are unclear, possible explanations include a changing environment, poor water quality, increased susceptibility to *E. rhusiopathiae* via emergence of a more pathogenic strain, or host immunosuppression in coastal bottlenose dolphins.

Links between environmental conditions and exposure *to E. rhusiopathiae* in other mammals have been found, although the mechanism is unclear (*5*). Similarly, short- and long-term ocean warming along the California coast could affect bacterial growth conditions or bottlenose dolphin prey. A change in prey could influence exposure if the presence, abundance, or pathogenicity of *E. rhusiopathiae* varies by fish species. In southern San Diego, untreated wastewater effluent from the Tijuana River Estuary and a wastewater treatment plant in Tijuana, Mexico, has resulted in poor ocean water quality and frequent beach closures (*6*). During a 2019–2020 winter study, *Erysipelothrix* spp. were detected by molecular genetic techniques in low numbers in the Tijuana River Estuary (*7*). However, it is unknown whether *E. rhusiopathiae* was present during the 2022 outbreak. It is also unknown whether coastal bottlenose dolphins have suppressed immune systems that may make them more susceptible to infection with *Erysipelothrix* spp. bacteria. Recorded concentrations of DDT compounds are higher among California coastal bottlenose dolphins than among any cetacean in the world (*8*), and halogenated organic compound load (e.g., from DDT) has been correlated with endocrine disruption in that population (*9*), which is relevant because endocrine function is closely tied to immune function (*10*).

If erysipelas outbreaks continue, they could threaten this relatively small population of dolphins. In addition, emergence of *E. rhusiopathiae* has potential health implications for persons who recreate in these waters or work with fish, and for free-ranging marine mammals or for other animals that prey on fish in this region.

Appendix Additional information from study of systemic erysipelas outbreak among free-ranging bottlenose dolphins, San Diego, California, USA, 2022.

## References

[1] Ugochukwu ICI, Samuel F, Orakpoghenor O, Nwobi OC, Anyaoha CO, Majesty-Alukagberie LO, et al. Erysipelas, the opportunistic zoonotic disease: history, epidemiology, pathology, and diagnosis—a review. Comp Clin Pathol. 2019;28:853–9. 10.1007/s00580-018-2856-5

[2] St. Leger J, Raverty S, Mena A. Cetacea. In: Terio KA, McAloose D, St. Leger J, editors. Pathology of Wildlife and Zoo Animals. Cambridge (MA): Academic Press; 2018. p. 533–68.

[3] Carretta JV, Oleson EM, Forney KA, Muto MM, Weller DW, Lang AR, et al. U.S. Pacific marine mammal stock assessments: 2021 [cited 2023 Oct 5]. https://repository.library.noaa.gov/view/noaa/44406

[4] IJsseldijk LL, Begeman L, Duim B, Gröne A, Kik MJL, Klijnstra MD, et al. Harbor porpoise deaths associated with *Erysipelothrix rhusiopathiae*, the Netherlands, 2021. Emerg Infect Dis. 2023;29:835–8. 10.3201/eid2904.221698 36958025 PMC10045706

[5] Aleuy OA, Anholt M, Orsel K, Mavrot F, Gagnon CA, Beckmen K, et al. Association of environmental factors with seasonal intensity of *Erysipelothrix rhusiopathiae* seropositivity among Arctic caribou. Emerg Infect Dis. 2022;28:1650–8. 10.3201/eid2808.212144 35876625 PMC9328914

[6] Feddersen F, Boehm AB, Giddings SN, Wu X, Liden D. Modeling untreated wastewater evolution and swimmer illness for four wastewater infrastructure scenarios in the San Diego-Tijuana (US/MX) border region. Geohealth. 2021;5:e2021GH000490. 10.1029/2021GH000490 PMC8581746 34796313

[7] Allsing N, Kelley ST, Fox AN, Sant KE. Metagenomic analysis of microbial contamination in the U.S. portion of the Tijuana River watershed. Int J Environ Res Public Health. 2022;20:600. 10.3390/ijerph20010600 36612923 PMC9819409

[8] Mackintosh SA, Dodder NG, Shaul NJ, Aluwihare LI, Maruya KA, Chivers SJ, et al. Newly identified DDT-related compounds accumulating in southern California bottlenose dolphins. Environ Sci Technol. 2016;50:12129–37. 10.1021/acs.est.6b03150 27737539 PMC6310127

[9] Trego ML, Hoh E, Whitehead A, Kellar NM, Lauf M, Datuin DO, et al. Contaminant exposure linked to cellular and endocrine biomarkers in southern California bottlenose dolphins. Environ Sci Technol. 2019;53:3811–22. 10.1021/acs.est.8b06487 30852886

[10] Wensveen FM, Šestan M, Turk Wensveen T, Polić B. ‘Beauty and the beast’ in infection: How immune-endocrine interactions regulate systemic metabolism in the context of infection. Eur J Immunol. 2019;49:982–95. 10.1002/eji.201847895 31106860

