# Supplementary material for: Systemic Erysipelas Outbreak among Free-Ranging Bottlenose Dolphins, San Diego, California, USA, 2022
Source: Emerg Infect Dis. 2023 Dec;29(12):2561–3. doi: 10.3201/eid2912.230811 (PMC10683814; doi:10.3201/eid2912.230811)
Supplement: Appendix — Additional information from study of systemic erysipelas outbreak among free-ranging bottlenose dolphins, San Diego, California, USA, 2022. [file 23-0811-Techapp-s1.pdf]

Article DOI: https://doi.org/10.3201/eid2912.230811

EID cannot ensure accessibility for supplementary materials supplied by authors. Readers who have difficulty accessing supplementary content should contact the authors for assistance.

Systemic Erysipelas Outbreak among Free-Ranging Bottlenose Dolphins, San Diego, California, USA, 2022

Appendix

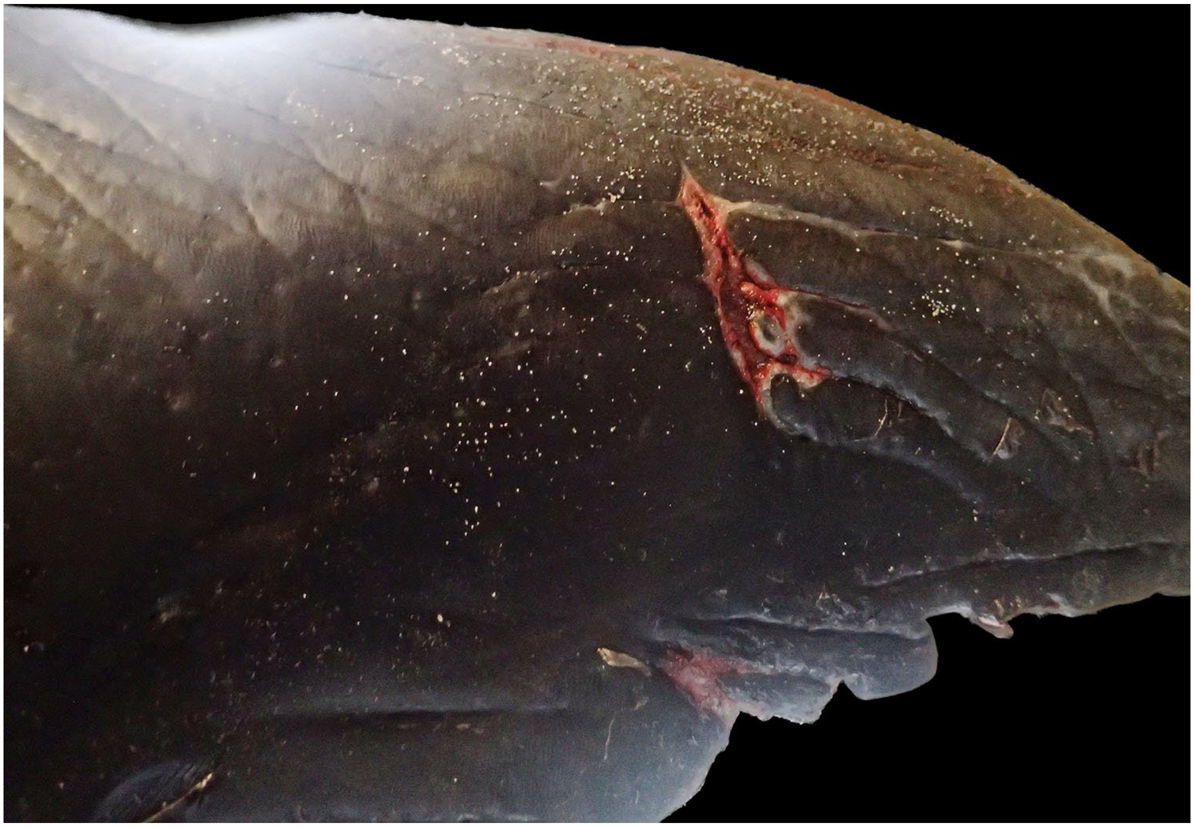

Appendix Figure. Fresh rake marks on flukes of *Tursiops truncatus* dolphin with systemic erysipelas, San Diego, California, USA, 2022.
